# miR-30b-3p通过靶向调控COX6B1抑制肺腺癌细胞的增殖和侵袭

**DOI:** 10.3779/j.issn.1009-3419.2022.101.42

**Published:** 2022-08-20

**Authors:** 琳 陈, 新璐 陈, 璐 刘, 燕乔 赵, 伟 左, 崇高 尹, 洪利 李

**Affiliations:** 1 261053 潍坊，潍坊医学院病理学教研室 Department of Pathology, Weifang Medical University, Weifang 261053, China; 2 261053 潍坊，潍坊医学院附属医院 Affiliated Hospital, Weifang Medical University, Weifang 261053, China; 3 261053 潍坊，潍坊医学院护理学院 Colloge of Nursing, Weifang Medical University, Weifang 261053, China; 4 261053 潍坊，潍坊医学院医学研究实验中心 Medical Research Center, Weifang Medical University, Weifang 261053, China

**Keywords:** 肺肿瘤, miR-30b-3p, 细胞色素C氧化酶6B1亚基, 增殖, 侵袭, Lung neoplasms, miR-30b-3p, COX6B1, Proliferation, Invasion

## Abstract

**背景与目的:**

肺腺癌（lung adenocarcinoma, LUAD）是当前最常见的肺癌亚型，微小RNA（microRNAs, miRNAs）是一类非编码小RNA，在细胞活动中发挥核心作用。miR-30b-3p在许多类型的癌症中均起到了关键作用，但关于其在肺腺癌中如何发挥作用的研究仍然很少。本研究通过探索miR-30b-3p在肺腺癌增殖和侵袭中的作用和机制，以期为临床上抑制肺腺癌的增殖和侵袭拓展新的方向。

**方法:**

利用NCBI生物数据库查找肺腺癌中差异表达明显的miRNA，并查询其差异表达及生存曲线；采用实时荧光定量聚合酶链反应（real-time fluorescence quantitative polymerase chain reaction, qRT-PCR）检测miR-30b-3p在各肺腺癌细胞系中的表达；5-乙炔基-2’脱氧尿嘧啶核苷（5-ethynyl-2'-deoxyuridine, EdU）细胞增殖实验和Transwell实验检测各组A549细胞增殖和侵袭能力的变化；使用在线预测数据网站确定miR-30b-3p的靶蛋白；Western blot验证COX6B1在不同组肺腺癌细胞中的表达情况；双荧光素酶实验证实miR-30b-3p与COX6B1是否存在结合位点。

**结果:**

miR-30b-3p在肺腺癌组织和细胞中表达下调（*P*<0.05），低表达水平的miR-30b-3p与肺腺癌患者的不良预后有关（*P*=0.005,8）；过表达miR-30b-3p能够抑制肺腺癌细胞的增殖与侵袭能力（*P*<0.05）；双荧光素酶实验证明miR-30b-3p和COX6B1存在结合位点（*P*<0.05）；Western blot实验表明在肺腺癌细胞A549中，过表达miR-30b-3p能够下调COX6B1的表达（*P*<0.05）；EdU细胞增殖实验和Transwell侵袭实验表明，miR-30b-3p的过表达能够逆转上调COX6B1对肺腺癌细胞增殖和侵袭能力的促进作用（*P*<0.05）。

**结论:**

miR-30b-3p在肺腺癌中起到了抑癌基因的作用，并能够通过调控COX6B1的表达来抑制肺腺癌的增殖和侵袭。

## Abstract (en)

**Background and objective:**

Lung adenocarcinoma (LUAD) is the most common clinical histological subtype of lung cancer and microRNAs (miRNAs) are a type of small non-coding RNAs which play a central role in cells. miR-30b-3p plays a key effect in many types of carcinoma, but there is still very little research on how it works in lung adenocarcinoma. The role and mechanism of miR-30b-3p in the proliferation and invasion of LUAD were explored in this study, to provide new targets for inhibiting the proliferation and invasion of LUAD.

**Methods:**

NCBI database was used to screen out miRNA with obvious differential expression, and the differential expression and survival curve were searched by StarBase database. Real-time fluorescence quantitative polymerase chain reaction (qRT-PCR) was used to detect the relative expression of miR-30b-3p in each lung adenocarcinoma cell line. 5-ethynyl-2'-deoxyuridine (EdU) cell proliferation assay and Transwell invasion assay were used to detect the proliferation and invasion of A549 cells in each group. The target genes of miR-30b-3p were determined by the target gene prediction websites. Western blot assay was used to detect the expression of COX6B1 in each group of A549 cells. Double luciferase assay was used to verify the targeted binding relationship between miR-30b-3p and COX6B1.

**Results:**

The expression of miR-30b-3p in lung adenocarcinoma tissues and lung adenocarcinoma cells was downregulated (*P*<0.05). Low expression levels of miR-30b-3p were associated with poor prognosis in patients with lung adenocarcinoma (*P*=0.005,8). Overexpression of miR-30b-3p could inhibit the proliferation and the invasion of lung adenocarcinoma cells (*P*<0.05). Double luciferase assay proved that miR-30b-3p could target and bind to COX6B1 (*P*<0.05). Western blot analysis showed that the overexpression of miR-30b-3p could downregulate the expression of COX6B1 in A549 cells (*P*<0.05). EdU cell proliferation assay and Transwell invasion assay showed that the overexpression of miR-30b-3p could reverse the promoting effect of upregulation of COX6B1 on proliferation and invasion in lung adenocarcinoma cells (*P*<0.05).

**Conclusion:**

miR-30b-3p acts as a tumor suppressor gene in lung adenocarcinoma, and it can inhibit the proliferation and invasion of lung adenocarcinoma by targeting the expression of COX6B1.

肺癌是目前全世界最多见的癌症死亡原因之一，其发病率每年呈上升趋势，是对全世界公众健康安全的严重挑战^[1]^。肺腺癌（lung adenocarcinoma, LUAD）是目前最为多见的肺癌病理类型^[2]^，其易出现远处转移，5年生存率较差。随着诊断、外科、放射、分子治疗等方面的不断进步，LUAD患者的临床治疗效果已经得到显著改善，但LUAD患者的早期诊断率不高，且在治疗过程中常易出现远处转移，5年生存率仍处于较低水平^[3, 4]^。因此，探究肺腺癌在人体如何发生发展的分子机制、寻求新的能够抑制肺腺癌增殖和侵袭的分子靶点格外重要。

微小RNA（microRNAs, miRNAs）是一类非编码蛋白质的，总体长约为23个核苷酸的小核糖核酸分子，其通过与互补的靶mRNA结合，在细胞生长、分化和个体发育中起着核心作用，能够调控mRNA的翻译抑制或降解^[5, 6]^。前期研究^[7, 8]^显示，miR-30b-3p在许多不同类型的癌症中均起到了关键作用。然而，miR-30b-3p与肺腺癌增殖和侵袭之间的关系仍未见详细报道。

本研究旨在探讨miR-30b-3p在肺腺癌增殖和侵袭中的作用和分子机制，以期为临床抑制肺腺癌的增殖和侵袭提供新的靶标。

## 1 材料与方法

### 1.1 细胞培养与材料

人正常肺支气管上皮细胞BEAS-2B与肺腺癌细胞A549、H1299均从ATCC获取，BEAS-2B细胞使用含有100 μL/mL FBS的DMEM高糖培养基，A549细胞和H1299细胞使用含有100 μL/mL FBS的RPMI-1640培养基，放于37 ℃、含5%CO_2_的细胞恒温培养箱中。Transwell小室购自Corning公司。Lipofectamine 2000购自Invitrogen公司。COX6B1（1:1, 000）、β-actin（1:1, 000）抗体均购自Abcam公司。

### 1.2 生物信息学数据预测

使用NCBI生物数据库（https://www.ncbi.nlm.nih.gov/）查找在肺腺癌和正常肺组织中具有表达差异的miRNA。使用StarBase数据库（https://starbase.sysu.edu.cn/index.php）分析miRNA在肺腺癌中的表达及其对肺腺癌患者预后生存的影响。

### 1.3 细胞转染

细胞培养方式均按照ATCC提供的培养条件进行。将A549细胞分组：①Con组：转染过表达miR-30b-3p质粒的对照质粒；②Over-miR-30b-3p组：转染过表达miR-30b-3p的质粒；③NC组：转染过表达COX6B1质粒的对照质粒；④Over-COX6B1组：转染过表达COX6B1的质粒；⑤Over-COX6B1+con组：同时转入过表达COX6B1的质粒和过表达miR-30b-3p质粒的对照质粒；⑥Over-COX6B1+over-miR-30b-3p组：同时转入过表达COX6B1的质粒和过表达miR-30b-3p的质粒。

### 1.4 实时荧光定量聚合酶链反应（real-time fluorescence quantitative polymerase chain reaction, qRT-PCR）

细胞中的总RNA提取及其逆转录具体过程可参照本课题组既往发表文献^[9]^。使用U6作为内参，使用2^-ΔΔCt^分析的方法对miR-30b-3p的表达进行分析。miR-30b-3p的上游引物为5’-AGGTGTTCAGCTGAGTGTAGG-3’，下游引物为5’-CAGTGCAGGGTCCGAGGT-3’，茎环结构为5’-GTCGTATCCAGTGCAGGGTCCGAGG TATTCGCACTGGATACGACCATCCT-3’。qRT-PCR设置条件为95 ℃、15 s，64 ℃、10 s，72 ℃、30 s，共反应42个循环。

### 1.5 5-乙炔基-2’脱氧尿嘧啶核苷（5-ethynyl-2'-deoxyuridine, EdU）细胞增殖实验

在各分组细胞中加入稀释好的1×EdU工作液，37 ℃培养箱孵育2 h后，用4%的多聚甲醛溶液固定细胞20 min，0.2%的Triton X-100通透细胞膜30 min，用配置好的Click反应液避光孵育30 min，使用Hochest染核。于正置荧光显微镜下随机选取不同区域拍照并对其计数。实验均独立重复3次。

### 1.6 Transwell侵袭实验

提前12 h将基质胶从-20 ℃取出于4 ℃冰箱过夜，稀释后取100 μL基质胶均匀涂抹于上室表面，37 ℃放置1 h，使其聚合成凝胶。小室下部滴加500 μL含100 μL/mL FBS的RPMI-1640培养基，小室中滴加含有4×10^4^个细胞的无血清RPMI-1640细胞悬液200 μL，37 ℃培养箱孵育24 h后，用细棉签轻轻擦去小室内部未穿膜的细胞，甲醇溶液固定细胞15 min，姬姆萨染液染色45 min。于显微镜下随机选取不同区域拍照并对其进行计数。实验独立重复3次。

### 1.7 在线网站预测靶蛋白

利用miRWalk（http://mirwalk.umm.uni-heidelberg.de/）、TargetScan（http://www.targetscan.org/）、miRTarbase（https://mirtarbase.cuhk.edu.cn/）网站，对miR-30b-3p进行靶蛋白预测，利用UALCAN（http://ualcan.path.uab.edu/index.html）、StarBase（https://starbase.sysu.edu.cn/index.php）、Kmplot（http://kmplot.com/analysis/index.php?p=background）网站对靶蛋白进行分析。

### 1.8 双荧光素酶报告基因实验

构建COX6B1的野生型质粒pGL3-COX6B1-3’-UTR-WT和突变型质粒pGL3-COX6B1-3’-UTR-MUT，将293T细胞接种于24孔板中，将miR-30b-3p的对照质粒及过表达质粒和COX6B1的野生型质粒、突变型质粒共转染入293T细胞中，48 h后加入PLB裂解液裂解细胞，使用双荧光素酶检测系统（Promega, Madison, USA）测定不同样品荧光素酶活性。

### 1.9 Western blot实验

将转染后的细胞用RIPA细胞裂解缓冲液分别提取总蛋白，应用BCA蛋白定量试剂盒对不同组细胞的蛋白浓度进行分析，加入5×上样缓冲液，沸水煮15 min。上样后，用12% SDS聚丙烯酰胺凝胶电泳分离蛋白，转膜，将转膜后的PVDF膜置于5%脱脂奶粉中于室温封闭1 h，后放入一抗中于4 ℃孵育8 h，然后在室温下滴加二抗孵育1 h，曝光。使用Image J对条带进行灰度值分析。

### 1.10 统计学方法

使用SPSS 23.0软件进行数据处理分析，计量结果使用均数±标准差（Mean±SD）表示，两组间比较使用*t*检验，多组间比较使用*F*检验。*P*<0.05表明差异具有统计学意义。

## 2 结果

### 2.1 在肺腺癌组织和细胞中低水平表达的miR-30b-3p与肺腺癌患者不良预后有关

通过对GSE19945数据集进行分析，筛选出log_2_FC<-1、*P*<0.05的miRNA，选择其中差异表达显著的miR-30b进行进一步分析研究（图 1A）。StarBase数据库显示，与20例正常肺组织相比，512例肺腺癌组织中miR-30b-3p的表达水平明显下降（图 1B，*P*<0.05）。通过StarBase数据库查询miR-30b-3p与肺腺癌患者预后的关系，结果显示，与miR-30b-3p高表达组相比，miR-30b-3p低表达组的生存率明显降低（图 1C，*P*=0.005,8）。这些数据证实，miR-30b-3p在肺腺癌中或许起到了抑癌基因的作用，低表达的miR-30b-3p与肺腺癌患者不良预后有关。我们为了证实这个结果，采用qRT-PCR检测了miR-30b-3p在肺支气管上皮细胞和肺腺癌细胞的表达，实验表明，与正常肺支气管上皮细胞BEAS-2B相比，miR-30b-3p在肺腺癌细胞A549和H1299中的表达量显著下调（图 1D，*P*<0.05）。因为miR-30b-3p在肺腺癌细胞A549中表达差异更为显著，所以选择肺腺癌细胞A549进行接下来的研究。

**图 1:**
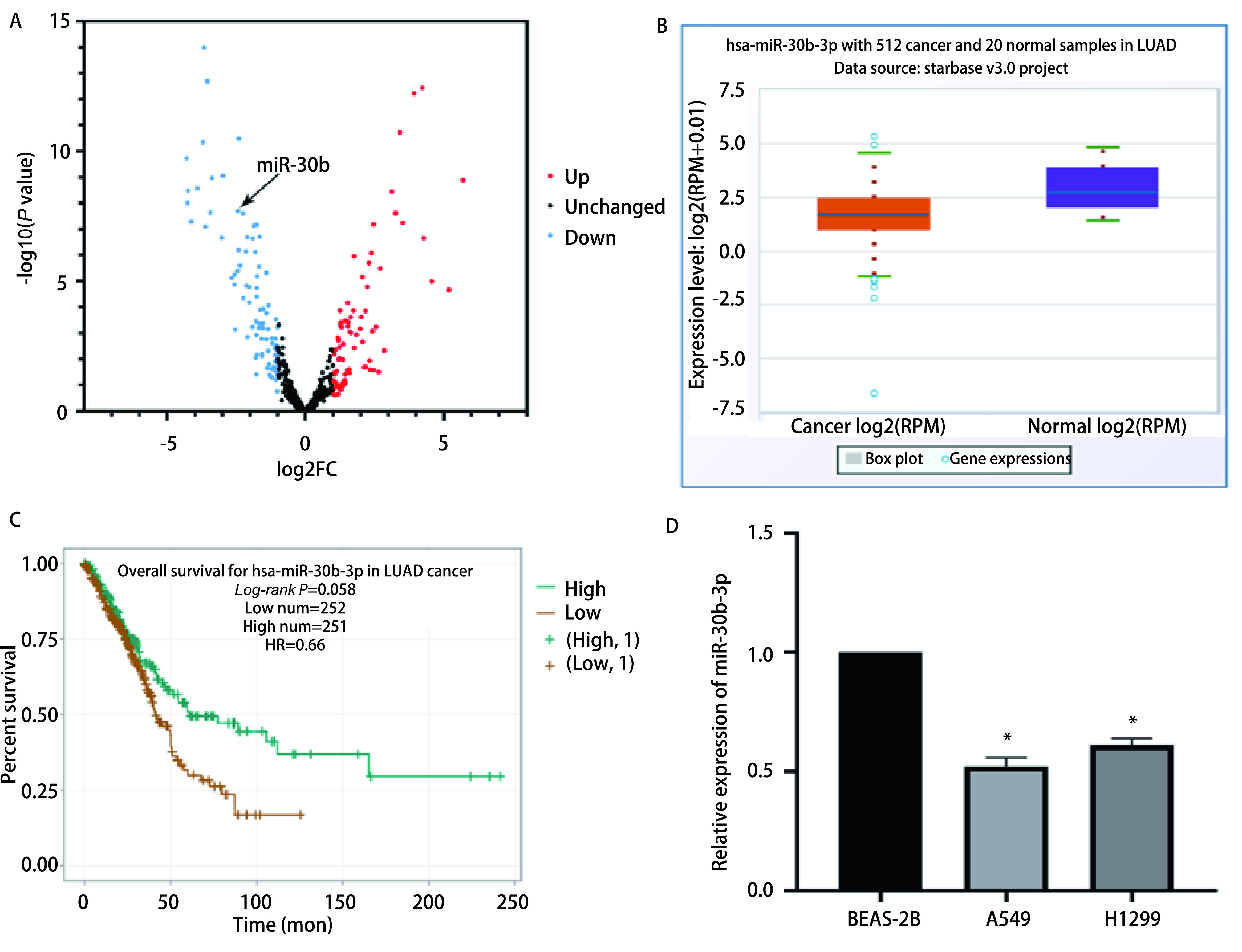
miR-30b-3p在肺腺癌组织和细胞中的表达及其与患者预后的关联程度。A：火山图显示miR-30b在数据集GSE19945中的表达差异；B：miR-30b-3p在正常肺组织和肺腺癌组织中的表达情况；C：miR-30b-3p的生存曲线分析；D：qRT-PCR验证miR-30b-3p在BEAS-2B、A549和H1299细胞系中的表达情况（^*^P<0.05）。 Expression of miR-30b-3p in lung adenocarcinoma tissues and cells and its association with patient prognosis. A: The volcano map of GSE19945; B: The expression of miR-30b-3p in normal lung tissues and lung adenocarcinoma tissues; C: The survival analysis of miR-30b-3p in lung adenocarcinoma patients; D: qRT-PCR analysis the expression of miR-30b-3p in BEAS-2B, A549 and H1299 cells (^*^P<0.05). qRT-PCR: real-time fluorescence quantitative polymerase chain reaction.

### 2.2 miR-30b-3p抑制肺腺癌细胞的增殖和侵袭能力

qRT-PCR实验证明，与对照组相比，转染过表达质粒over-miR-30b-3p后A549细胞中miR-30b-3p的表达量明显上升（图 2A，*P*<0.05），表明过表达成功。通过EdU细胞增殖实验验证其对A549细胞增殖能力的影响，与转染对照组质粒的A549细胞（Con/A549组）相比，转染过表达质粒的A549细胞（Over-miR-30b-3p/A549组）的EdU阳性率显著下降（图 2B，*P*<0.05），这证实了过表达miR-30b-3p后，肺腺癌细胞的增殖能力出现显著下调。Transwell侵袭实验表明，与Con/A549组相比，Over-miR-30b-3p/A549组穿过基质胶的细胞数量明显下降（图 2C，*P*<0.05），提示过表达miR-30b-3p可以抑制肺腺癌细胞的侵袭能力。综上所述，我们发现miR-30b-3p可以抑制肺腺癌细胞的增殖与侵袭能力。

**图 2:**
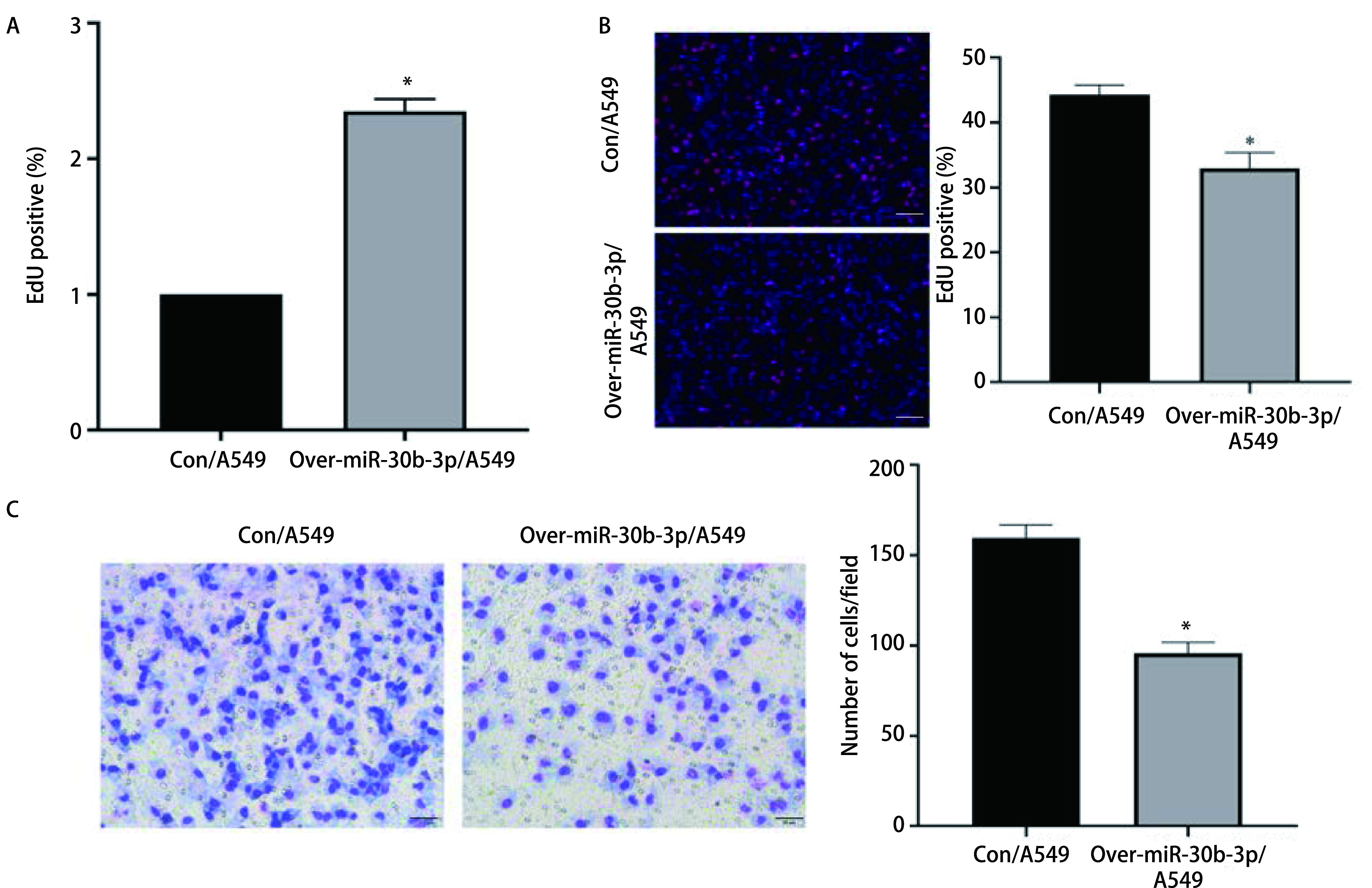
miR-30b-3p抑制肺腺癌细胞的增殖和侵袭能力。A：qRT-PCR验证过表达质粒miR-30b-3p的转染效率（^*^P<0.05）；B：EdU实验验证上调miR-30b-3p对肺腺癌细胞增殖能力的影响（×20）（^*^P<0.05）；C：Transwell实验验证上调miR-30b-3p对肺腺癌细胞侵袭能力的影响（×20）（^*^P<0.05）。 miR-30b-3p inhibited the proliferation and invasion of lung adenocarcinoma cells. A: qRT-PCR analysis detected the transfection efficiency of miR-30b-3p overexpressed plasmid (^*^P<0.05); B: EdU experiment detects the effect of up-regulation of miR-30b-3p on the proliferation ability of lung adenocarcinoma cells (×20), scale bar=100 *μ*m (^*^P<0.05); C: Transwell experiment detects the effect of up-regulation of miR-30b-3p on the invasion ability of lung adenocarcinoma cells (×20), scale bar=50*μ*m (^*^P<0.05). EdU: 5-ethynyl-2'-deoxyuridine.

### 2.3 miR-30b-3p能够与COX6B1靶向结合

利用靶基因预测网站miRWalk、TargetScan、miRTarbase预测可与miR-30b-3p结合的靶蛋白，取交集得26个靶蛋白（图 3A）。利用StarBase网站和UALCAN网站对26个靶蛋白进行分析，其中COX6B1在肺腺癌组织中高表达（图 3B，*P*<0.05），并且其表达和miR-30b-3p的表达呈负相关（图 3C，*r*=-0.200，*P*<0.05）。利用Kmplot数据库查询COX6B1与肺腺癌患者预后的关系，结果显示高表达水平的COX6B1与患者的不良预后有关，且差异具有统计学意义（图 3D，*P*<0.05）。因此我们选取COX6B1进行下一步研究。利用Western blot实验验证COX6B1在肺支气管上皮细胞BEAS-2B与肺腺癌细胞A549中的表达情况，结果表明，与BEAS-2B细胞相比，COX6B1在A549细胞中的表达明显上调（图 3E，*P*<0.05）。设计双荧光素酶实验拟证实miR-30b-3p与COX6B1之间是否能够直接结合，结果显示，COX6B1 mRNA的3’-UTR区是miR-30b-3p的直接结合位点（图 3F，*P*<0.05）。Western blot实验检测Con/A549组和Over-miR-30b-3p/A549组中COX6B1的表达情况，可见与Con/A549组相比，Over-miR-30b-3p/A549组中COX6B1的表达量大幅下降（图 3G，*P*<0.05），这表明在肺腺癌细胞A549中，miR-30b-3p可以负向调控COX6B1的表达。以上结果表明，COX6B1 mRNA的3’-UTR区是miR-30b-3p的直接结合位点，且miR-30b-3p能够负向调控COX6B1的表达。

**图 3:**
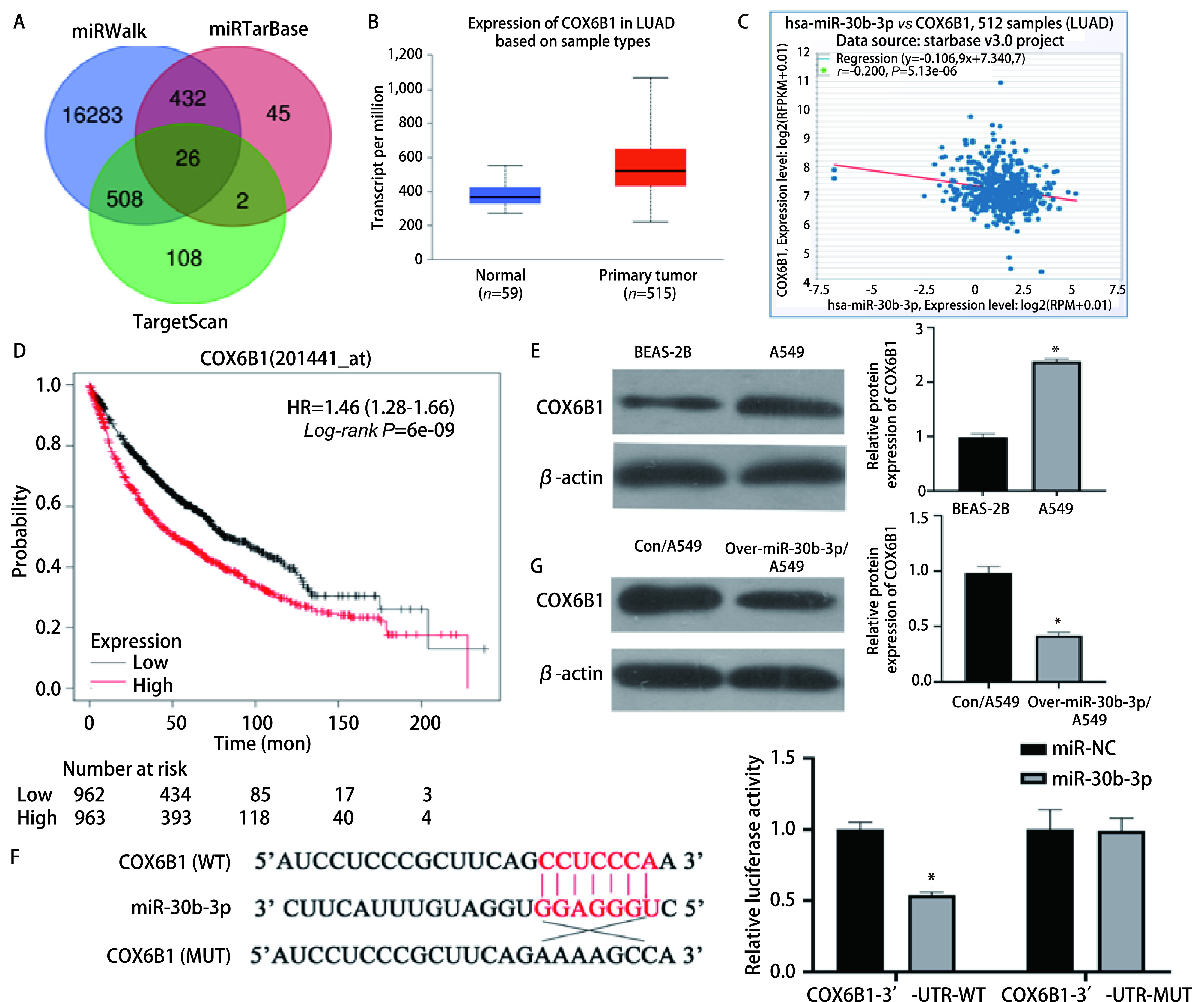
miR-30b-3p靶向结合COX6B1。A：基因预测维恩图；B：COX6B1在正常肺组织和肺腺癌组织中的不同表达；C：miR-30b-3p和COX6B1的相关性分析；D：COX6B1的生存曲线分析；E：Western blot验证COX6B1在BEAS-2B细胞和A549细胞中的表达情况（^*^P<0.05）；F：双荧光素酶实验验证miR-30b-3p对COX6B1的靶向结合能力（^*^P<0.05）；G：Western blot实验验证过表达miR-30b-3p后COX6B1的表达情况（^*^P<0.05）。 miR-30b-3p targeted COX6B1. A: The venn plot of predicted genes; B: The expression of COX6B1 in normal lung tissues and lung adenocarcinoma tissues; C: Co-Expression analysis for miR-30b-3p with COX6B1; D: The survival analysis of COX6B1; E: Western blot analysis showed the expression of COX6B1 in BEAS-2B cells and A549 cells (^*^P<0.05); F: Luciferase activity was used to detect the targeted binding ability of miR-30b-3p to COX6B1 (^*^P<0.05); G: Western blot to detect the expression of COX6B1 after overexpression of miR-30b-3p (^*^P<0.05).

### 2.4 miR-30b-3p通过调控COX6B1抑制肺腺癌细胞的增殖能力

为了证明miR-30b-3p和COX6B1存在调控关系，我们构建了COX6B1的过表达质粒转染至A549细胞中，Western blot实验结果表明，与NC/A549组相比，Over-COX6B1/A549组中COX6B1的表达明显升高（图 4A，*P*<0.05），表明过表达COX6B1成功。EdU细胞增殖实验结果显示，与NC/A549组相比，Over-COX6B1/A549组的细胞增殖率显著上升，而与Over-COX6B1+con/A549组相比，Over-COX6B1+over-miR-30b-3p/A549组的细胞增殖率明显下降（图 4B，*P*<0.05）。这表明上调COX6B1可以促进A549细胞的增殖能力，而过表达miR-30b-3p可以逆转上调COX6B1对细胞增殖能力的促进作用。以上结果证实，miR-30b-3p可以通过负向调控COX6B1抑制肺腺癌细胞A549的增殖能力。

**图 4:**
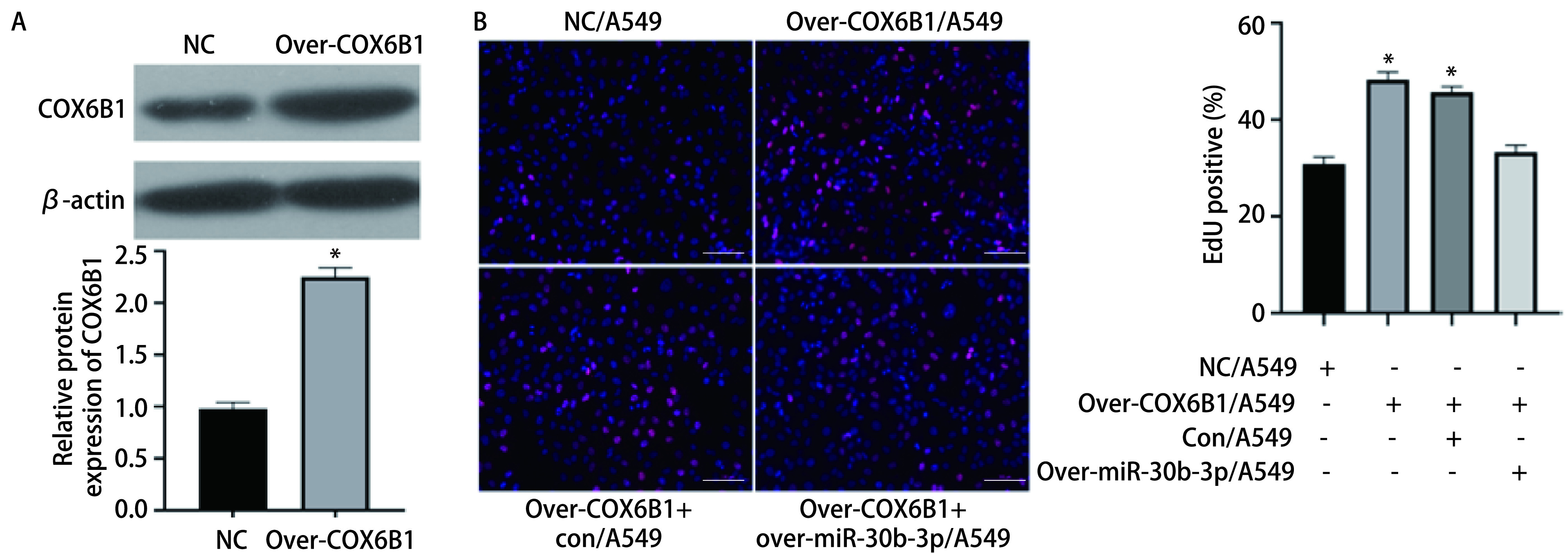
miR-30b-3p靶向调控COX6B1抑制肺腺癌细胞的增殖能力。A：Western blot实验验证COX6B1的过表达情况（^*^P<0.05）；B：EdU实验检测miR-30b-3p与COX6B1共转染对肺腺癌细胞增殖能力的影响（×20）（^*^P<0.05）。 miR-30b-3p inhibited the proliferation of lung adenocarcinoma cells by targeting COX6B1. A: Western blot analysis showed the expression of COX6B1 in different groups of cells (^*^P<0.05); B: EdU experiment detects the effect of miR-30b-3p and COX6B1 co-transfection on the proliferation ability of lung adenocarcinoma cells (×20), scale bar=100 *μ*m (^*^P<0.05).

### 2.5 miR-30b-3p通过调控COX6B1抑制肺腺癌细胞的侵袭能力

Transwell侵袭实验验证miR-30b-3p靶向结合COX6B1对A549细胞侵袭能力造成的影响。与NC/A549组相比，Over-COX6B1/A549组穿过基质胶的细胞数量显著增加，而与Over-COX6B1+con/A549组相比，Over-COX6B1+over-miR-30b-3p/A549组穿过基质胶的细胞数量明显减少（图 5，*P*<0.05），表明上调COX6B1可以促进A549细胞的侵袭能力，而同时过表达miR-30b-3p可以逆转上调COX6B1对A549细胞侵袭能力的促进作用。以上结果提示，miR-30b-3p可以通过负向调控COX6B1抑制肺腺癌细胞A549的侵袭能力。

**图 5:**
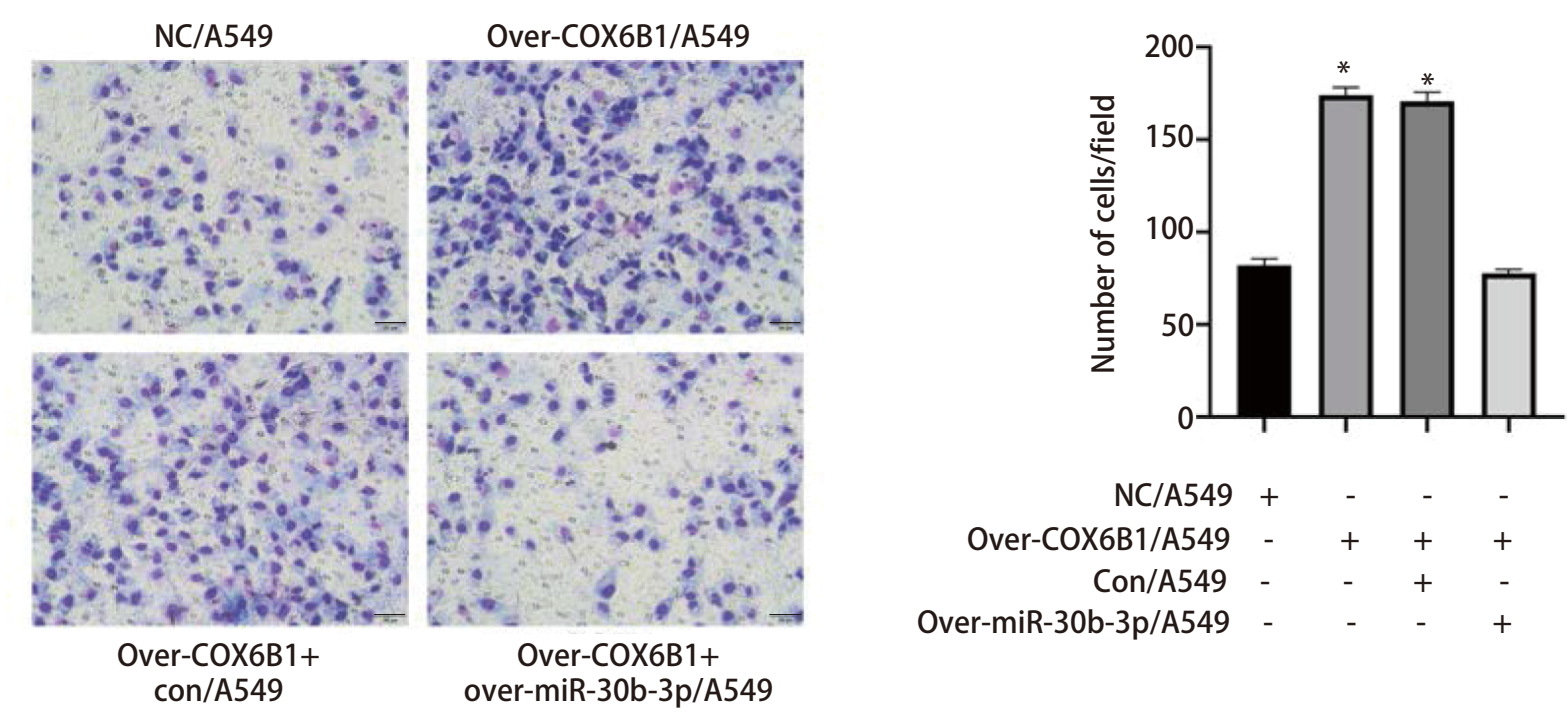
miR-30b-3p靶向调控COX6B1抑制肺腺癌细胞的侵袭能力（×20）（^*^P<0.05） miR-30b-3p inhibited the invasion of lung adenocarcinoma cells by targeting COX6B1 (×20), scale bar=50 *μ*m (^*^P<0.05)

## 3 讨论

肺癌是目前全世界最为常见的癌症种类之一，它是全球癌症相关死亡的主要原因，其中近年来肺腺癌的发病率在全球范围内持续上升^[10]^。由于肺腺癌的早期诊断和治疗的手段并不多见，探究肺癌进展的潜在分子机制及其分子治疗靶点显得尤为重要^[11]^。因此，寻找肺腺癌分子发病机制中的特征性靶点是提高其早期诊断率和提升患者生存质量的关键所在。

miRNA是一类非编码RNA，因其长度短小而得名，通常有18个-22个核苷酸长度。既往研究^[12, 13]^表明，大多数（至少60%）的蛋白编码基因可以被miRNA调控。单个的miRNA可以同时靶向几十个，甚至上百个基因。由于miRNA在调节基因表达中的关键作用，其表达失调或功能异常常见于各种类型的疾病，包括心血管异常、自身免疫紊乱、阿尔茨海默病和各种类型的恶性肿瘤^[14-17]^。近年来，有报道称部分miRNA在肺癌中表达降低，与此相互印证的是，部分miRNA的降低也与肺癌患者的不良预后有关。例如，miR-218通过靶向IL-6/STAT3在肺癌中发挥抑癌作用^[18]^，miR-192抑制肺癌细胞增殖并诱导细胞凋亡^[19]^等。既往报道^[20, 21]^中，miR-30b-3p可以抑制卵巢癌以及肝癌的细胞增殖或侵袭，但miR-30b-3p在肺腺癌中发挥了怎样的作用却仍未可知。在本研究中，通过NCBI数据库和StarBase数据库发现，miR-30b-3p在肺腺癌组织和细胞中表达降低，并且低表达水平的miR-30b-3p与肺腺癌患者的不良预后存在一定相关性，这些都提示miR-30b-3p的异常表达可能和肺腺癌存在一定关联，因此确定了miR-30b-3p作为研究对象。本研究通过qRT-PCR分析证明，与正常肺支气管上皮细胞BEAS-2B相比，miR-30b-3p在肺腺癌细胞系H1299和A549中的表达均大幅降低。此外，体外细胞实验证明，miR-30b-3p能够下调肺腺癌细胞的增殖能力和侵袭能力，在肺腺癌中起到了抑癌基因的作用。

*COX6B1*基因位于19q13号染色体上，是细胞色素C氧化酶的亚基之一，在多种组织和细胞系中都有表达，对于细胞呼吸链起着不可或缺的作用，并且参与了细胞的氧化磷酸化等过程^[22]^。有研究^[23, 24]^表明，COX6B1在肾透明细胞癌、子宫内膜癌等恶性肿瘤中都发挥了作用，然而COX6B1与肺腺癌是否存在关系尚未见报道。本文通过在线网站查询发现，COX6B1在肺腺癌组织中表达大幅升高，并且其与患者的不良预后相关。双荧光素酶实验结果证明，COX6B1是miR-30b-3p的靶点，miR-30b-3p可与COX6B1 mRNA的3’-UTR结合。Western blot实验证明，COX6B1在肺腺癌细胞中表达上调，并且过表达miR-30b-3p可以抑制COX6B1在肺腺癌细胞中的表达。EdU细胞增殖实验和Transwell侵袭实验证明，miR-30b-3p的过表达能够逆转上调COX6B1对肺腺癌细胞增殖能力和侵袭能力的促进作用。以上研究表明，miR-30b-3p可以通过负向调控COX6B1抑制肺腺癌细胞A549的侵袭和增殖能力。本文通过研究miR-30b-3p与COX6B1的相互作用，为完善miR-30b-3p与肺腺癌的关系提供了理论参考，为肺腺癌治疗提供了新的方向。

综上所述，miR-30b-3p在肺腺癌组织和细胞中表达下调，低表达水平的miR-30b-3p与肺腺癌患者不良预后有关；miR-30b-3p可以通过负向调节COX6B1，从而抑制肺腺癌细胞的增殖和侵袭能力。此外，miR-30b-3p调控COX6B1的分子机制尚不得而知，而如何将其与临床相结合仍尚需进一步探讨，这也将成为我们接下来探索与研究的重点。
